# Healing vs. hurting words: the power of language in pain perception—a narrative review

**DOI:** 10.3389/fpain.2026.1835194

**Published:** 2026-06-08

**Authors:** Giulia Leonardi, Francesco Bonanno, Angelo Alito, Amerigo Stamile, Carmela De Domenico, Antonio Di Dio, Francesca Sposito, Carmen Cucinotta, Adriana Tisano, Simona Portaro

**Affiliations:** 1 Department of Physical and Rehabilitation Medicine, University Hospital “G. Martino”, Messina, Italy; 2 Department of Clinical and Experimental Medicine, University of Messina, Messina, Italy; 3 Department of Biomedical, Dental Sciences and Morphological and Functional Images, University of Messina, Messina, Italy; 4 Brain Mapping Lab, Department of Biomedical, Dental Sciences and Morphological and Functional Imaging, University of Messina, Messina, Italy; 5 Outpatient Specialist, Provincial Health Authority of Cosenza, Cosenza, Italy; 6 IRCCS Centro Neurolesi Bonino Pulejo, Messina, Italy

**Keywords:** clinical communication, nocebo effect, pain perception, patient-centered care, therapeutic alliance, word processing

## Abstract

Pain is increasingly understood as a multidimensional and inferential experience shaped not only by nociceptive input but also by cognitive, emotional, and contextual factors. Among these modulators, language appears to play a central role in shaping pain perception through semantic framing, verbal suggestion, expectation, and interpersonal communication. This narrative review aimed to synthesise contemporary evidence on the relationship between language and pain perception, with particular focus on musculoskeletal rehabilitation and Pain Neuroscience Education (PNE). A structured literature search was conducted in PubMed and Scopus for English-language studies published between 2016 and 2026. Experimental studies, randomized controlled trials, neuroimaging investigations, and clinically oriented rehabilitation studies examining linguistic influences on pain perception were considered eligible. Sixteen studies met inclusion criteria and were narratively synthesised. Three interconnected domains emerged from the evidence. First, pain was consistently conceptualised as an inferential and expectation-shaped experience, in which attention, prior beliefs, and predictive processes modulate sensory interpretation. Second, verbal suggestion and semantic framing were shown to influence pain-related neural and behavioural responses. Pain-related words, threatening descriptors, and negative expectations were associated with increased pain perception and altered activity within salience and pain-regulatory networks, whereas positive framing and empathic communication appeared to facilitate adaptive modulation and placebo-related responses. Third, PNE and language-based rehabilitation approaches demonstrated potential to reduce fear, catastrophising, and maladaptive threat appraisal, particularly when integrated within multimodal rehabilitation programs including exercise, motivational interviewing, and psychologically informed physiotherapy. Overall, the evidence suggests that language is not merely descriptive but functions as an active contextual and neurocognitive component of pain experience. Clinically, communication may influence therapeutic alliance, behavioural engagement, and pain-related beliefs, with both therapeutic and ethical implications. However, current evidence remains limited by methodological heterogeneity, short-term experimental paradigms, and limited ecological validity. Language-based interventions should therefore be considered facilitators within broader biopsychosocial rehabilitation strategies rather than stand-alone treatments. Future longitudinal and clinically pragmatic studies are needed to clarify the durability, mechanisms, and translational applicability of linguistic modulation in pain rehabilitation.

## 1 Introduction

Pain Is defined by the International Association for the Study of Pain (IASP) as “an unpleasant sensory and emotional experience associated with actual or potential tissue damage or described in terms of such damage” (1). This definition highlights that pain is not just a sensory signal, but rather a complex phenomenon influenced by biological, cognitive, emotional, and contextual factors.

From a neurobiological perspective, pain arises when nociceptors are activated and processed within a distributed network involving spinal cord, thalamus, insula, anterior cingulate cortex, and prefrontal regions (2, 3). This network, historically described as the “pain matrix”, is now more accurately conceptualised as a dynamic system that integrates bottom-up nociceptive input with top-down cognitive, emotional and contextual signals. Thus, rather than representing a fixed anatomical entity, it reflects interacting neural processes that continuously shape the subjective experience of pain (4, 5).

Within this framework, symbolic and semantic stimuli, including language, appear to function as powerful top-down modulators. Experimental evidence suggests that words and verbal framing can influence the activity of pain-related neural circuits and, consequently, the subjective experience of pain, even in the absence of changes in peripheral nociceptive input (6, 7). Emotional factors such as expectations, attention, and affective states further modulate pain processing by amplifying or dampening neural responses to sensory input (8).

Importantly, many of these modulatory processes are established during clinical interactions. Verbal framing acts as a contextual signal that shapes expectations and threat appraisal, thereby influencing both pain perception and behavioural responses (9–11).

Language is a particularly effective contextual trigger because it activates semantic and emotional processing systems that interact with core pain modulatory networks. Consequently, words can influence how pain is constructed and experienced, as well as simply describing it (12).

Within predictive processing frameworks, pain is increasingly described as an inferential process under uncertainty, whereby the nervous system continuously integrates prior beliefs with incoming sensory information (8). In this context, pain-related language provides top-down information that may influence prediction error signalling within insular and cingulate regions, thereby shaping perception and appraisal. These models are primarily theoretical frameworks that integrate converging empirical findings, rather than definitive mechanistic accounts, and should be interpreted accordingly (13, 14).

Negative or threatening verbal cues tend to increase pain perception, whereas reassuring, empathic communication appears to reduce pain intensity and distress. In this sense, language may behave analogously to a pharmacological agent, triggering placebo or nocebo effects during clinical encounters (15, 16).

Neurobiological studies suggest that verbally induced placebo and nocebo responses may involve activation of endogenous opioid and dopaminergic systems, as well as modulation of salience and pain-regulatory networks, including the anterior cingulate cortex, insula, and periaqueductal grey (17, 18). It is important to note that these mechanisms appear to be context-dependent and subject to significant variability between individuals (19).

In rehabilitation settings, Pain Neuroscience Education (PNE) represents a structured clinical application of language aimed at rewriting patients' understanding of pain. By reframing pain as a protective yet potentially modifiable output of the nervous system, PNE seeks to reduce fear, catastrophising, and exaggerated threat appraisal (20, 21). Although substantial evidence supporting the effects of language and expectation on pain processing has emerged, this literature remains fragmented across the fields of neuroscience, psychology, linguistics and rehabilitation research (22).

Moreover, experimental findings are rarely synthesised in a way that directly informs everyday communication strategies in musculoskeletal rehabilitation. Linguistic, cognitive, and contextual mechanisms are often examined in isolation, limiting their translation into clinical practice. Accordingly, there is a need for a narrative synthesis that integrates these perspectives with a specific focus on musculoskeletal pain and rehabilitation. This narrative review therefore aims to synthesise current evidence on the role of language in pain perception by conceptualising verbal communication as (1) a cognitive stimulus shaping expectations and attention, (2) a neural modulator influencing pain-related brain networks through top-down mechanisms, and (3) a therapeutic tool intentionally employed in clinical interventions.

## 2 Methods

A structured literature search was conducted in the PubMed and Scopus databases to identify peer-reviewed studies published in English that explored the relationship between language and pain perception. The final search was performed in March 2026. The search strategy focused on literature published between 2016 and 2026 to capture contemporary developments in pain neuroscience, communication research, placebo/nocebo mechanisms, and rehabilitation approaches.

The PubMed Boolean search strategy included combinations of the following terms: (“pain” OR “pain perception” OR “nociception”) AND (“language” OR “communication” OR “semantics” OR “verbal suggestion” OR “framing” OR “word processing” OR “pain neuroscience education”). Equivalent search combinations were adapted for Scopus. Filters were applied for human studies and English-language publications.

Studies were considered eligible if they: (a) investigated linguistic or communicative influences on pain perception, including verbal suggestion, semantic priming, framing, clinician–patient communication, or educational approaches; (b) involved human participants with musculoskeletal pain or experimentally induced pain relevant to rehabilitation and clinical contexts; and (c) were designed as randomised controlled trials or experimental studies published in peer-reviewed journals. Exclusion criteria included animal studies, non-English publications, conference abstracts, editorials, studies not related to word processing in pain management, and studies outside the rehabilitation field.

The search identified 579 records from PubMed and 836 records from Scopus, resulting in a total of 1,415 records screened based on title and abstract. During the initial screening process, records were excluded for the following reasons: publication before 2016 (*n* = 594), non-randomised or non-experimental design (*n* = 261), non-English language (*n* = 26), lack of relevance to word processing in pain management (*n* = 150), lack of relevance to rehabilitation settings (*n* = 128), and duplicate records (*n* = 227). After screening, 29 full-text articles were assessed for eligibility. Full-text exclusions included records not retrieved (*n* = 3), irrelevant settings (*n* = 4), inadequate or inappropriate sample size (*n* = 2), and ongoing or incomplete studies (*n* = 4)**.** Ultimately, 16 studies met all inclusion criteria and were included in the final synthesis.

Screening and study selection were independently conducted by two reviewers, with disagreements resolved through discussion and consensus among the research team.

Given the heterogeneity of study designs, populations, linguistic stimuli, and outcome measures, a quantitative synthesis was not considered appropriate; findings were therefore synthesised narratively. To further strengthen the methodological quality assessment, the risk of bias of the included randomised controlled trials was evaluated using the Cochrane Risk of Bias 2 (RoB 2) tool (23), whereas non-randomised and experimental studies were assessed using the Newcastle–Ottawa Scale (NOS) (24). For studies employing non-randomised experimental designs or mechanistic approaches (e.g., neuroimaging, computational modelling or theoretical frameworks), we applied a structured narrative appraisal framework that addressed six methodological domains: (1) clarity of the research question and articulation of the mechanism, (2) appropriateness of the methodological design for the research question, (3) quality of the data and transparency of reporting, (4) directness and clinical relevance, (5) internal consistency and plausibility, and (6) replicability. Each domain was scored on a 0–2 scale (low, moderate or high), and an overall certainty rating (high, moderate, low or very low) was assigned. This approach ensures a transparent assessment of the quality of the evidence while accommodating design heterogeneity and enabling quality-graded findings to be integrated into the narrative synthesis. Framework development integrated principles from SANRA, GRADE, Cochrane methodology, and domain-specific standards for neuroimaging and experimental pain research.

## 3 Results

Across the included literature, findings converged on three interrelated domains describing how language, cognition, and pain interact: (1) pain and cognition, (2) pain and verbal suggestion, and (3) pain neuroscience education and clinical translation. While presented separately for clarity, the evidence consistently suggests a continuum from cognitive inference to communicative modulation and, ultimately, clinical reconceptualization. The included evidence comprised heterogeneous study designs, including experimental laboratory paradigms, neuroimaging investigations, randomized clinical trials, qualitative studies, and theoretical models. Most experimental studies were conducted in healthy volunteers and evaluated short-term effects, limiting ecological validity and generalisability to routine clinical practice.

Table 1 provides a comprehensive summary of the key findings from the included studies, organised by thematic clusters.

**Table 1:** Synthesis of evidence from the included studies.

Domain	Study	Study design	Population/sample	Intervention/exposure	Main outcomes	Principal findings	Key limitations
Pain & cognition	Onysk et al. (25)	Experimental computational modelling study	Healthy participants	Statistical learning paradigm with painful stimuli	Pain prediction and pain intensity ratings	Expectations and confidence independently modulated pain perception, supporting inferential models of pain processing.	Experimental laboratory setting; uncertain clinical applicability.
	Lier et al. (26)	Experimental cognitive study	Healthy participants	Attention-demanding cognitive tasks during painful stimulation	Pain perception during attentional manipulation	Attentional engagement may reduce pain perception, although effects were context- and task-dependent.	Considerable variability in effects; short-term paradigm only.
Verbal suggestion & neurobiology	Borelli et al. (12)	Neuroimaging study	Healthy volunteers	Exposure to pain-related semantic stimuli	Brain activation patterns	Pain-related words partially shared neural substrates with nociceptive pain, particularly in salience and affective networks.	Correlational neuroimaging findings; no direct clinical outcomes.
	Mauchand et al. (31)	Experimental neurocognitive study	Healthy participants	Emotional prosody in spoken pain complaints	Neural empathic and salience responses	Vocal emotional tone modulated empathic neural responses independently of semantic content.	Limited ecological validity.
	Ritter et al. (10)	Experimental pain study	Healthy volunteers	Pain-related vs. neutral verbal cues before noxious stimulation	Pain ratings and neural activation	Negative pain-related words increased pain ratings and altered pain-related neural processing.	Healthy volunteer sample; uncertain durability of effects.
	Vaegter et al. (33)	Randomized controlled experimental trial	Healthy participants	Positive vs. negative verbal information before exercise	Exercise-induced hypoalgesia and pain thresholds	Expectations induced through verbal information modulated hypoalgesic responses following exercise.	Short-term effects only; limited external validity.
	Swannell et al. (32)	Experimental semantic priming study	Healthy volunteers	Exposure to pain-related words	Pain ratings and laser-evoked potentials	Semantic activation of pain concepts increased subjective pain ratings and neurophysiological responses.	Experimental paradigm without clinical follow-up.
	Pazzaglia et al. (30)	Experimental neurophysiological study	Healthy participants	Verbal suggestion regarding expected pain intensity	Habituation of pain ratings and laser-evoked potentials	Negative expectation disrupted physiological habituation to pain stimuli.	Small sample size and short observation period.
	Bartels et al. (27)	Randomized experimental trial	Healthy humans	Positive verbal suggestion with conditioning paradigm	Nocebo response modulation	Positive suggestion combined with conditioning appeared to reduce nocebo responses.	Findings derived from experimental rather than clinical settings.
	Brodhun et al. (29)	Experimental behavioural study	Healthy participants	Acute pain exposure with word valence assessment	Emotional evaluation of pain-related words	Pain altered emotional evaluation of pain-related language, suggesting bidirectional interactions.	Acute pain model only.
	Yazbek et al. (28)	Cross-sectional linguistic study	Patients with low back pain	Linguistically adapted pain descriptors	Pain reporting accuracy	Linguistically congruent descriptors improved communication and pain reporting.	Context-specific cultural sample limits generalisability.
PNE & clinical translation	Kasimis et al. (34)	Randomized clinical trial	Individuals with chronic non-specific low back pain	Pain neuroscience education + motivational interviewing + manual therapy	Pain and disability outcomes	Multimodal intervention produced greater improvements in pain and disability.	Language-specific contribution difficult to isolate.
	Vicente-Mampel et al. (37)	Randomized controlled trial	Elderly individuals in community physiotherapy	Pain neuroscience education programme	Pain perception and psychosocial variables	PNE alone produced modest improvements with heterogeneous psychosocial responses.	Limited long-term follow-up.
	Nijs et al. (35)	Clinical framework/practical guide	Chronic pain rehabilitation context	Integration of motivational interviewing within PNE	Behavioural engagement and communication strategies	Combining explanatory language with motivational communication may facilitate behavioural change.	Primarily conceptual and clinically oriented paper.
	Wijma et al. (38)	Clinical biopsychosocial framework paper	Chronic pain populations	Biopsychosocial assessment framework	Clinical communication and contextual assessment	Emphasised beliefs, communication, and contextual meaning in chronic pain management.	Theoretical framework without primary empirical data.
	Mittinty et al. (36)	Interventional observational study	Chronic pain patients	Pain education intervention	Recovery expectations and pain intensity	Cognitive reconceptualization following pain education was associated with lower pain and improved recovery expectations.	Association-based findings; causal mechanisms uncertain.

PNE, pain neuroscience education.

### 3.1 Pain and cognition: pain as an inferential, expectation- and attention-shaped experience

Across cognitive and mechanistic accounts, pain emerges not as a passive registration of nociception but as an interpreted perceptual experience. Research on expectations and predictive processing indicates that prior beliefs bias pain perception by shaping how sensory information is weighted.

Computational modelling suggests that the nervous system continuously updates pain predictions based on feedback, with confidence influencing how strongly new information revises prior beliefs (25). Attention-based modulation has been widely explored through distraction paradigms, with cognitively demanding tasks often reducing perceived pain. However, the magnitude and reliability of these effects vary considerably, indicating that context, task characteristics, and individual differences shape outcomes (26). Collectively, this body of evidence positions pain as a meaning-laden experience in which expectations, attentional priorities, and learned associations determine what is perceived as threatening and therefore painful.

### 3.2 Pain and verbal suggestion: semantic content, framing, prosody, and neurobiological correlates

The evidence suggests that language is a multidimensional construct with distinct communicative functions relating to pain. Semantic framing provides the cognitive “what” of the interaction, and specific pain-related words or threatening descriptors may act as semantic primers, increasing sensitivity and activating affective-sensory networks (12, 27, 28). Importantly, this relationship appears bidirectional: pain experiences can also influence the emotional evaluation of pain-related language, suggesting shared affective substrates (29, 30).

Emotional prosody and vocal expression determine how empathic responses and perceived importance are modulated, regardless of the literal word choice. These findings suggest that, although framing affects the content of patients' expectations, prosody and interpersonal tone influence the emotional safety of clinical interactions (31, 32). Neuroimaging studies provide mechanistic support for these effects, showing that verbal cues about expected pain intensity modulate activity and connectivity within regulatory networks implicated in placebo analgesia (10, 33). However, these findings should be interpreted cautiously, as most evidence derives from experimental paradigms and correlational neuroimaging studies rather than direct demonstrations of causal clinical effects. At the same time, many studies rely on short-term laboratory paradigms in healthy volunteers, limiting ecological validity and raising questions about durability and generalisability.

### 3.3 Pain neuroscience education and clinical translation

The third domain addresses how language-based approaches, particularly PNE, are applied clinically. PNE aims to reconceptualise pain as a protective but modifiable nervous system output. Meta-analytic and theoretical work supports its potential to reduce pain-related disability, particularly when integrated with active rehabilitation (34, 35).

Intervention studies suggest that PNE may be more effective when combined with exercise, manual therapy, or motivational interviewing, indicating that language and movement may converge on shared recovery pathways (36). Nevertheless, response variability is substantial, and education alone does not reliably produce clinically meaningful change for all individuals. This highlights the importance of personalised, biopsychosocial implementation.

Relational and cultural factors further modulate outcomes. Empathy, communication style, and metaphor use influence engagement and distress, and linguistic framing may have unintended nocebo effects depending on cultural meaning and personal history (37, 38). Regarding the extent of clinical outcomes, the evidence indicates a clear pattern: while PNE as a standalone intervention often results in modest reductions in pain intensity, integrating it into multimodal programs produces more significant improvements in pain and functional disability. This is most evident in patients with chronic musculoskeletal pain characterised by high levels of fear avoidance and catastrophising (34–36).

### 3.4 Quality of the studies included

Methodological quality assessment demonstrated overall moderate methodological quality across the included studies. Randomized controlled trials generally presented low risk in randomization and outcome reporting domains, whereas some concerns emerged regarding blinding procedures and deviations from intended interventions due to the behavioural and educational nature of the interventions. Non-randomized studies achieved moderate-to-good NOS scores, particularly in participant selection and outcome assessment domains, although comparability between groups was inconsistently controlled. Detailed quality assessments are summarized in Tables 2, 3.

**Table 2:** Cochrane RoB 2 assessment of randomized controlled trials.

Study	Randomization process	Deviations from intended interventions	Missing outcome data	Measurement of outcome	Selection of reported results	Overall risk of bias
Vaegter et al. (33)	Low risk	Unclear	Low risk	Low risk	Low risk	Unclear
Bartels et al. (27)	Low risk	Unclear	Low risk	Low risk	Low risk	Unclear
Kasimis et al. (34)	Low risk	Unclear	Low risk	Unclear	Low risk	Unclear
Vicente-Mampel et al. (37)	Low risk	Unclear	Low risk	Unclear	Low risk	Unclear

**Table 3:** Newcastle–Ottawa scale (NOS) assessment of Non-randomized studies.

Study	Selection (max 4)	Comparability (max 2)	Outcome/exposure (max 3)	Total score	Quality
Yazbek et al. (28)	3	1	2	6/9	Fair
Brodhun et al. (29)	3	1	3	7/9	Good
Pazzaglia et al. (30)	3	1	2	6/9	Fair
Mittinty et al. (36)	4	1	3	8/9	Good

Five neuroimaging and behavioral experimental studies (10, 12, 25, 31, 32) demonstrated high methodological quality with transparent reporting, appropriate statistical corrections, and replicable methods, though most employed restricted samples (healthy participants, female-only cohorts). One behavioral experimental study (26) achieved moderate-to-high quality with solid two-experiment replication design but ceiling effects in cognitive accuracy measures. Two clinical framework papers (35, 38) provided evidence synthesis rather than primary empirical data and were classified as moderate quality for clinical guidance applications. An overview of the analysis is provided in Table 4.

**Table 4:** Methodological quality assessment of eight representative studies across six evaluative domains. Studies are stratified by design type and overall quality tier (high, moderate-to-high, moderate).

Study	Design	Q1	Q2	Q3	Q4	Q5	Q6	Overall
Mauchand et al. (31)	fMRI neuroimaging	High	High	High	Moderate	High	High	High
Borelli et al. (12)	fMRI neuroimaging	High	High	High	Moderate	High	High	High
Ritter et al. (10)	fMRI + behavioral	High	High	High	Moderate	High	Moderate	High
Swannell et al. (32)	Behavioral + EEG	High	High	High	Moderate	High	Moderate	High
Onysk et al. (25)	Computational modeling	High	High	High	Moderate	High	Moderate	High
Lier et al. (26)	Experimental behavioral	High	High	Moderate	High	Moderate	Moderate	Moderate-to-high
Nijs et al. (35)	Clinical framework synthesis	Moderate	N/A	Moderate	High	Moderate	Moderate	Moderate
Wijma et al. (38)	Clinical framework synthesis	Moderate	N/A	Moderate	High	Moderate	Moderate	Moderate

N/A, not applicable; Q1, research question clarity; Q2, methodological appropriateness; Q3, data quality & reporting; Q4, directness & clinical relevance; Q5, internal consistency; Q6, replicability.

## 4 Discussion and clinical application

The synthesis suggests that pain is not solely a biological alarm but a meaning-driven experience shaped by inference, communication, and context (39). Cognitive and computational models support the view that expectations and learning influence how sensory information is interpreted and therefore influence pain (40). These interpretations are also consistent with broader biopsychosocial and enactive models of pain, which conceptualise pain as an embodied and context-sensitive experience shaped by behavioural, emotional, and interpersonal factors (41, 42)**.** However, much of this evidence derives from controlled paradigms with limited ecological validity.

Neurocognitive studies demonstrate that expectations modulate pain-related processing via changes in prediction error signalling within cortical and frontostriatal networks, supporting the view of pain as an inference under uncertainty (8, 9). Importantly, predictive processing models should be interpreted as theoretical and integrative frameworks rather than definitive mechanistic explanations of pain.

Experimental studies demonstrate that words and framing can modulate pain sensitivity and pain-related neural processing, yet durability of these effects in clinical populations remains uncertain.

The clearest clinical translation is provided by PNE, which seeks to shift appraisal, reduce threat, and restore agency (20, 37). Outcomes appear more robust when PNE is embedded within multimodal rehabilitation, although substantial inter-individual variability persists. This is consistent with previous systematic reviews suggesting that pain neuroscience education may be most effective when integrated with active rehabilitation and psychologically informed approaches rather than delivered as a stand-alone intervention (40, 43, 44). It is important to note that PNE should not be implemented in isolation as a cognitive intervention. Therefore, the evidence points to a dual-level component. From a neurobiological perspective, language may contribute to modulation of neural circuits involved in pain and attention. However, clinically, it acts as a facilitator that reduces threat perception, creating a “window of opportunity” for behavioral engagement and physical rehabilitation (10, 12, 25). In clinical terms, positive words, and educational reframing act as a top-down primer, reducing the nervous system's resistance to movement. However, to ensure long-term durability and address the limitations of short-term experimental paradigms, this cognitive shift should be reinforced through behavioural exposure and active rehabilitation. This interpretation aligns with fear-avoidance and psychologically informed rehabilitation models, which emphasise that changes in beliefs and expectations should be paired with corrective behavioural and sensory experiences (42, 45, 46)**.**

Language is the primary means of fostering the therapeutic alliance and regulating affect. However, the distinction between “healing” and “hurting” words is not just a matter of clinical style; it is also a difference in neurobiological processes. While supportive and empathic communication has been associated with activation of endogenous pain modulatory systems in experimental placebo research, threatening or negatively framed language may contribute to nocebo-related responses (6, 12). Evidence suggests that pain-related semantic stimuli share common neural substrates with actual nociceptive processing (10, 32). However, these observations derive primarily from laboratory and neuroimaging paradigms and should not be interpreted as evidence that specific words directly produce tissue-level or deterministic clinical effects. This demonstrates that threatening words can physically engage the same affective-sensory networks as tissue damage. Consequently, negative framing (e.g., “bone-on-bone” or “degenerative changes”) does not merely describe a condition, but can actively increase pain sensitivity and disrupt physiological habituation (12). This concern is supported by evidence showing that clinician communication can have enduring effects on pain beliefs, reassurance, and behavioural engagement (47–49). At present, the extent and durability of these effects in routine clinical practice remain insufficiently established. In an attachment-based framework, this kind of communication is seen as a high-threat signal, which increases prediction error signaling in the insula (8, 18). This, in effect, makes the nervous system more sensitive rather than calm. This shows that potentially harmful language may carry ethical implications, as it can reinforce perceptions of bodily vulnerability and contribute to disability-related beliefs (50).

Similarly, contextual and relational factors are increasingly recognised as clinically relevant modulators of placebo and nocebo responses in musculoskeletal rehabilitation (51–53). Examples of potentially nocebogenic phrases include statements such as “your spine is degenerating,” “your back is unstable,” or “this condition will only get worse.” These may be associated with increased fear and threat appraisal. In contrast, alternative formulations such as “these imaging findings are common and not necessarily related to pain,” “your spine is strong and adaptable,” or “movement can help improve function over time” may better support adaptive expectations while preserving clinical accuracy. These elements raise ethical considerations: communication can shape pain experience rather than merely describe it, placing responsibility on clinicians to use language thoughtfully and context-sensitively.

The clinical efficacy of language is most evident in patients with chronic musculoskeletal pain, particularly when it is used to reduce high levels of fear, catastrophizing and exaggerated threat appraisal (20, 21). In terms of clinical outcomes, evidence suggests that language-based interventions, when used in isolation, may only achieve modest reductions in pain intensity (9, 12). Emerging approaches such as Cognitive Functional Therapy and psychologically informed physiotherapy further support the integration of communication, behavioural exposure, and personalised meaning-making within rehabilitation (45, 46).

Furthermore, the strategic use of language is particularly important in telerehabilitation and remote clinical settings. In digital environments where physical touch is absent, verbal communication is the main way of building a secure working relationship and providing therapeutic advice (43, 54, 55).

However, integrating language into multimodal rehabilitation involving exercise, manual therapy, or motivational interviewing significantly amplifies the therapeutic impact, leading to greater improvements in both pain and disability.

Personalisation is therefore critical. Within this framework, reassurance should not be considered a uniform intervention. Its effectiveness depends on how it is delivered and contextualised. Reassurance is most beneficial when it is credible, individualised, and explicitly combined with validation of the patient's experience. Conversely, overly simplistic or dismissive reassurance may inadvertently contribute to increased uncertainty, reduced trust, or heightened distress (44). Reassurance may be particularly useful in the early stages of assessment, when patients present with high levels of fear, uncertainty, or catastrophic interpretations of symptoms. However, it may be less effective or even counterproductive when used as a substitute for explanation, functional guidance, or active rehabilitation strategies.

As a narrative review, this work does not follow a formal systematic protocol or quantitative quality assessment, which may introduce selection bias. Study designs, linguistic stimuli, and outcome measures varied widely, and most research involved Western, English-speaking populations. Long-term effects of linguistic interventions remain insufficiently studied. An additional limitation concerns the methodological heterogeneity of the included literature. Furthermore, several of the included studies relied on mechanistic laboratory paradigms, neuroimaging experiments, computational models, or theoretical frameworks, which could not be formally assessed using RoB 2 or NOS criteria due to their non-standardized methodological designs. Although these studies provide relevant neurobiological and explanatory insights, their ecological validity and direct clinical applicability remain limited.

Importantly, the evidence reviewed does not support the interpretation of positive or empathic language as an isolated therapeutic intervention. Rather, pain neuroscience education, semantic reframing, and therapeutic communication should be understood as components of a broader multimodal rehabilitation approach, integrated with exercise therapy, behavioural strategies, and patient-centred care. This perspective is consistently maintained throughout the manuscript to avoid overinterpretation of linguistic effects as standalone therapeutic mechanisms.

Despite these findings, concerns remain about the durability and ecological validity of language effects. Much of the current neurobiological evidence may not fully capture the complexities of persistent pain. Consequently, the long-term sustainability of verbally induced neural modulation remains insufficiently studied. In clinical practice, language should be considered a therapeutic primer that reduces the nervous system's resistance to movement. However, this cognitive shift must be reinforced with active, sensory-based activities, such as exercise, to prevent the effects from being temporary.

Ultimately, biopsychosocial assessment and behavioral intervention are connected by language. It is not the only way to bring about change, but it does help people to think differently, which means that later physical interventions are possible. By integrating language into a wider framework of patient-centered care, clinicians can employ framing and prosody to develop a shared narrative that aligns the patient's existing beliefs with the rehabilitation therapy's objectives.

Multimodal approaches aligning physiological modulation with reconceptualization and behaviour change may further enhance outcomes. Overall, language may not heal in isolation, but -by structuring meaning- it can influence appraisal, behaviour, and neurobiological regulation. Future research should prioritise pragmatic, real-world designs that examine durability, cultural context, and moderators of response.

## 5 Conclusions

Words matter. They can amplify pain or alleviate it by shaping meaning, expectations, and neural processing. Evidence from neuroscience and rehabilitation research indicates that language is not a neutral medium but an active component of pain experience. Within clinical practice, this implies that communication may have therapeutic and ethical consequences. Integrating linguistically informed approaches into rehabilitation offers a low-cost and scalable opportunity to support therapeutic alliance, reduce maladaptive threat appraisal, and facilitate engagement in active rehabilitation. However, language-based interventions should not be considered stand-alone treatments and appear most clinically meaningful when integrated within multimodal, biopsychosocial rehabilitation approaches. Future research should prioritize methodologically robust longitudinal clinical studies capable of integrating mechanistic neurobiological findings with ecologically valid rehabilitation outcomes, thereby improving the translational applicability of language-based interventions in pain management.
